# Suitability of Placental Blood Samples of Newborns for Pre-Transfusion Testing

**DOI:** 10.3389/fped.2021.661321

**Published:** 2021-04-30

**Authors:** Rana Alissa, Patty D. Williams, Erika L. Baker, Jennifer A. Hipp, Jinous Saremian, Agnes E. Aysola

**Affiliations:** ^1^Department of Pediatrics, University of Florida College of Medicine Jacksonville, Jacksonville, FL, United States; ^2^Department of Pathology, University of Florida College of Medicine Jacksonville, Jacksonville, FL, United States

**Keywords:** placental blood, heel stick, pre-transfusion testing, less invasive, natural

## Abstract

**Objective:** To show concordance between heel stick and placental blood sample pairs for newborns' pre-transfusion testing and to validate placental blood's tube and gel methodology.

**Methods:** Placental samples were collected for pre-transfusion testing at birth from 78 singleton and twin newborns admitted to our Mother–Baby Unit to compare with the results of heel stick samples taken from same newborns. Gestational age ≥35 weeks, weight ≥2,000 g. The study was approved by the Institutional Review Board (IRB). Informed consent was obtained from newborn parents. ABO blood group, Rhesus factor (Rh), direct antiglobulin test (DAT), and antibody screen were performed. Ortho ProVue Analyzer was used for tube and gel methods. McNemar's test for paired categorical data was performed.

**Results:** One hundred percent concordance in 78 pairs for ABO and Rh. Seventy-four pairs were tested for antibodies, 72 were both negative, 1 was both positive, and 1 gave discordant result. Ninety-nine percent concordance, *p* = 0.999. Sixty-five pairs were both DAT negative, seven were both DAT positive, and six gave discordant results. Ninety-two percent concordance, *p* = 0.68. Placental blood gave identical results comparing tube with gel methods.

**Conclusions:** Placental blood is suitable for pre-transfusion testing and can replace heel sticks. Placental blood tube and gel methods are validated.

## Introduction

Heel stick is the main source of blood testing in term and premature newborns. It is an invasive and painful procedure that affects newborn's future developmental and behavioral expressions. It also has long-term effects on their pain response when compared to infants who did not have such an invasive procedure (1, 2). Furthermore, in 2004, a study showed a specific association between parental concerns about infant's pain and parental distress (3).

While there are many studies focusing on minimizing the pain of infants during heel sticks (4), there has been much interest in preventing any invasive procedures on newborns altogether. One example was the creation of the sepsis risk calculator (5), which has minimized the need for workup in infants at risk for early onset sepsis. Another example is the use of non-invasive transcutaneous bilirubin which has replaced infant's serum bilirubin when screening for neonatal jaundice (6).

The placenta has been at the center of some of these interests. It is the only organ in the human body that expires and gets disposed into the medical biohazard waste after the birth of an infant. Numerous recent studies suggest that before it is thrown away, the placental blood can be useful and can replace infant's blood for various testing.

Our group, Aysola et al. (7), concluded in a recent publication in 2018 that the placenta can replace the infant's blood for pre-transfusion testing. In our study, premature infants admitted to the Neonatal Intensive Care Unit (NICU) underwent comparison between paired placental blood and heel stick blood that resulted in 100% concordance in all pairs. Therefore, in our hospital, the placental blood was validated to be used for pre-transfusion testing using the gel and tube methods in infants <35 weeks gestational age (GA) and <2,000 g weight. However, this study excluded infants who were ≥2,000 g in weight and ≥35 weeks GA, and it also excluded twins.

In contribution to finding other ways to test infants accurately without the need to invasively collect blood from them, we conducted this study, which is the second phase of the previous study by Aysola et al. (7). This second-phase study aims to validate the placental blood for pre-transfusion testing for singleton and twin newborns ≥35 weeks GA and ≥2,000 g weight. It also aims to validate the tube and gel methodology for placental blood samples.

## Materials and Methods

This study was conducted in the Mother–Baby Unit (MBU) at the University of Florida in Jacksonville. Our MBU admits infants who are ≥35 weeks GA and ≥2,000 g weight. The study is done as a collaboration between the Department of Pediatrics and the Department of Pathology, specifically the blood bank, at our institution.

It was submitted and approved by the IRB in February 2019. All parents of qualified singleton and twin infants who were admitted to the MBU were approached for consideration to be enrolled in this study. Qualified infants whose parents signed a consent form were enrolled in the study. Infants admitted to the MBU, but whose parents declined enrollment in the study, were excluded.

Infants who had parental consents had paired blood samples collected. These paired samples were placental samples collected within 30 min of birth, and heel stick samples collected during any time prior to the infant's discharge from the hospital. The heel stick collection for the sake of this study was done only in combination with other tests such as glucose and bilirubin, if needed, or the newborn screen. The blood samples collected from the placenta or from the infant were taken immediately to the blood bank for appropriate processing and testing.

**Figure 1:**
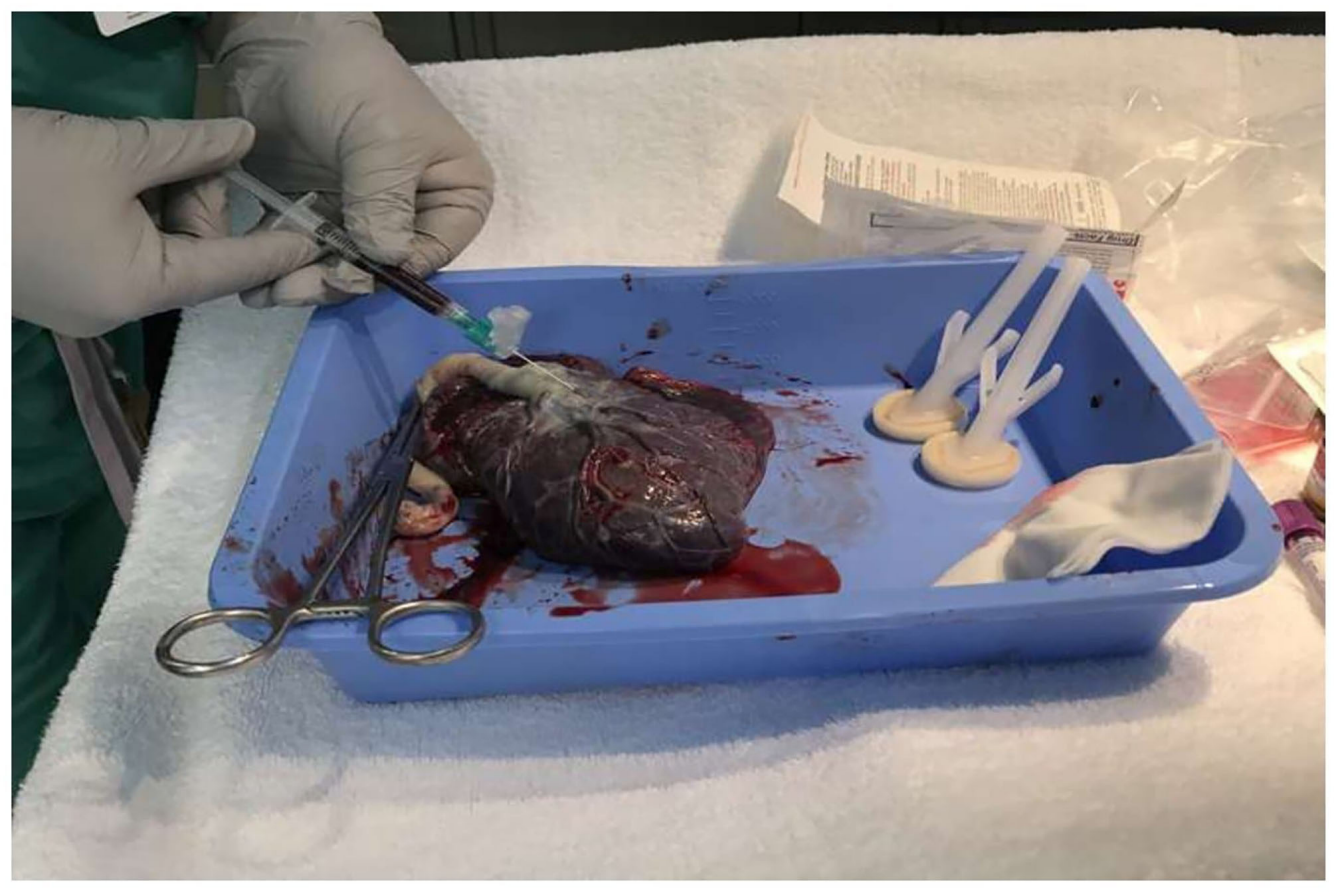
Collecting blood sample from placenta.

Placental blood samples were collected after cleaning the placental surface area surrounding the base of the cord insertion (Figure 1).

The cleaning process included drying the area twice using 4 × 4 sterile gauze, then sterilizing it with chlorhexidine preparation, and finally drying the wet area again with another 4 × 4 sterile gauze. Placental venous blood (1–2 ml) is then collected using a 21-gauge needle and a 3-ml syringe. The blood collected was immediately transferred to a 4-ml EDTA purple tube, appropriately labeled, and then taken to the blood bank for processing and testing.

We used anti-A, -B, and -D reagents from ALBAclone (Quotient Biodiagnostics, Newtown, PA, USA) to perform ABO and Rh typing in the tube. For the antibody screen, we used Selectogen I and II cells; the cell concentration in the tube was 3 ± 1%, while it was 0.8 ± 0.2% with gel technology. We used Ortho Antigram 0.8% Resolve Panel for antibody identification, while for DAT testing, we used anti-IgG (Rabbit). The screening cells for antibody screen and identification, as well as the ID-MTS gel cards, were all by Ortho Clinical Diagnostics. The technologist decided whether gel tests were done manually or by the automated instrument ProVue (Ortho Clinical Diagnostics).

ABO and RH typing and direct antiglobulin test with IgG (DAT) were performed on the sample pairs. Antibody screen tests were performed on 74 of these sample pairs, since four cases had insufficient volume for antibody screen. Both tube and gel methods were used to validate both methods for the placental samples; the gel tests were performed by an Ortho ProVue^®^ Analyzer. Statistical analysis was performed using exact McNemar's test for paired categorical data, using SAS^®^ for Windows version 9.4 (8).

## Results

This study shows 100% concordance in 78 sample pairs for ABO and Rh test results (Table 1).

**Table 1:** ABO and RH test results.

**ABO Rh results**	**Placental samples *n* = 78**	**Heel stick samples *n* = 78**	**ABO type frequency in study**
O positive	31	31	40%
A positive	26	26	33%
B positive	16	16	21%
AB positive	1	1	1%
A negative	3	3	4%
B negative	1	1	1%

Of the 74 sample pairs tested for the presence of an antibody, 72 sample pairs were both negative and one sample pair was both positive, giving 99% concordance rate.

Only 1 (1%) sample pair gave a discordant result. This occurred when the placental sample was positive for the presence of an antibody, while the heel stick sample was negative; *p* = 0.999 for discordance. Interestingly, the positive antibody results were 1+ positive with both screening cells. In contrast, in the case of the discrepant result, only one of the screening cells gave a 1+ positive result with the placental sample.

Regarding DAT test results, 65 (83%) sample pairs were both negative, while seven (9%) of the sample pairs were both DAT positive, giving 92% concordance rate for DAT test (Table 2). Four (5%) of the placental sample tested DAT positive, while the corresponding heel stick sample gave negative results. Two (3%) of the heel stick samples were DAT positive, while the placental samples were negative. Statistical analysis shows the lack of discordance between the sample pairs regarding DAT results (*p* = 0.68).

**Table 2:** DAT test results.

**DAT results**	**Placental samples *n* = 78**	**Heel stick samples *n* = 78**
Negative	67	69
Positive	11	9
**Discordant rate: 8% (*n* = 6)**		
Positive	4	2
Negative	2	4

In 10 of the 11 DAT-positive cases, when either the placental sample or the heel stick sample were positive, maternal anti-A or anti-B antibodies coating newborns' red cells were responsible for the positive DAT result. In the one remaining case, both mother and infant had B positive blood type and the antibody screen of the mother's serum was negative. We hypothesize that non-specific IgG antibodies crossing the placenta from maternal circulation gave the positive DAT result in this case.

There were 14 twins enrolled: 2 monochorionic-diamniotic and 12 dichorionic-diamniotic.

## Discussion

Relying on the placental blood as an accurate alternative to the infant's blood for testing purposes is the goal for many health advocates. These advocates are calling for a natural, low-risk, and safe newborn period that is free from invasive procedures such as heel sticks.

In 2017, a case report regarding the use of placental/umbilical blood sampling (PUBS) suggested that PUBS identified pathogens even when the critically ill infant's direct blood was negative (9). In 2018, multiple publications were discussed comparing the placental venous blood to the infant's blood for complete blood count (CBC) and blood culture testing. Greer et al. concluded that placental venous blood is suitable for obtaining CBC and differential and it is also an appropriate second source for blood culture with appropriate placental surface cleaning techniques to avoid contamination (10). Newberry found that placental blood to test CBC and blood cultures of infants at all gestational ages served as a reliable source of blood when compared to paired blood collected from infants who were at risk for early-onset sepsis (11). Additionally, on day 1 of the Section on Neonatal–Perinatal Medicine Program meeting at the American Academy of Pediatrics, placental venous blood usage was discussed for neonates being admitted to the NICU. It was concluded that placental venous blood was suitable for obtaining both a CBC and differential and as a second source for blood culture as it yields additional true pathogens (10).

Another important reason to find other accurate methods or sources to test infants other than their own blood is iatrogenic anemia that may result from diagnostic blood loss. Iatrogenic anemia is a topic that is often discussed in pediatrics especially in the Intensive Care Units (ICUs) when pediatric patients are premature or critically ill or both (12, 13). Also, anemia in the donor twin in Twin to Twin Transfusion Syndrome (TTTS) is a serious complication in the monochorionic diamniotic twin gestation (14). Although the indication for blood transfusion in term and late preterm infants is significantly less than premature infants, it is still prevalent. Examples include but are not limited to placental hemorrhage, umbilical cord abnormalities, subgaleal hemorrhage, hemolytic anemia, and hemorrhagic disease of the newborn especially in the light of increased parental refusal of administering vitamin K to their newborns (15–18). In all these clinical scenarios, every drop of blood count is significant.

All of the above in addition to the focus on the long-term effects of neonatal pain from many researchers make this study very important. Despite the use of non-pharmacological strategies to reduce neonatal pain in our MBU during the heel stick procedure such as sweet-tasting substances, kangaroo care, breastfeeding, formula feeding, non-nutritive sucking, and swaddling (19), neonatal pain remains underestimated and undertreated due to lack of pain assessment tools in this field. As prior neonatal experience may influence current pain experience or the risk of persistent pain, all this should be considered within the biopsychosocial assessment and formulation of pain development (20).

Part 1 of our study was able to validate the placental blood for pre-transfusion testing and for the gel method; however, this was done only in premature infants who were admitted to NICU and excluded infants who were admitted to MBU and twins (7). The current study, Part 2, included twins and infants who were admitted to MBU, and it achieved similar results.

Limitations of our study include the following: (1) Negative rhesus blood type infant's sample was small but compatible with the general populations. (2) Smaller number of twin delivery in our institution during the study period than desired for the study. (3) The majority of the twins were Di-Di with no Mono-Mono and only one Mono-Di.

The results of this study validated our hypothesis that placental blood samples are suitable for pre-transfusion testing and can replace heel stick samples to reduce blood sample collection from newborns. This study also validated the gel method for ABO and Rh typing of placental blood samples. Based on the results of this study, our blood bank accepts placental blood samples for pre-transfusion testing for term and twins infants. Future studies to further compare the placental blood to infant's blood are needed, especially when significant amount of blood is warranted for accurate testing. A few examples of these tests include screening for syphilis, human immunodeficiency virus, toxoplasmosis, and chromosomal microarray. This would result in our ultimate goal of minimum to no invasive blood draws during a newborn's hospital stay after delivery.

## Data Availability Statement

The original contributions presented in the study are included in the article/supplementary material, further inquiries can be directed to the corresponding author/s.

## Ethics Statement

The studies involving human participants were reviewed and approved by Institutional Review Board Ethics Committee. Written informed consent to participate in this study was provided by the participants' legal guardian/next of kin.

## Author Contributions

RA and AA were responsible for designing the study, writing the protocol, and reviewed the full manuscript. AA, JS, and RA were responsible for the IRB submission process. RA, PW, and EB screened, consented, and collected the blood from qualified infants for the study. AA supervised the testing, interpreted the results, extracted and analyzed the data, and wrote the results section. RA wrote the body of the manuscript. JH edited the final manuscript. All authors contributed to the article and approved the submitted version.

## Conflict of Interest

The authors declare that the research was conducted in the absence of any commercial or financial relationships that could be construed as a potential conflict of interest.
